# Redistributing working schedules using the infective principle in the response to COVID-19

**DOI:** 10.1017/ice.2020.155

**Published:** 2020-04-21

**Authors:** Marko Ćurković, Andro Košec, Petrana Brečić

**Affiliations:** 1 University Psychiatric Hospital Vrapče, Zagreb, Croatia; 2 School of Medicine, University of Zagreb, Zagreb, Croatia; 3 Department of Otorhinolaryngology and Head and Neck Surgery, University Hospital Center Sestre milosrdnice, Zagreb, Croatia

*To the Editor*—Many healthcare professionals are currently witnessing, or being actively involved in dealing with, the COVID-19 global pandemic and its tragic pathways and outcomes.^1,2^ The battle against this pandemic is putting enormous pressure on healthcare systems worldwide, primarily due to the infectiousness of the infective agent and the specific health consequences it causes.^2,3^ Most public health measures are aimed at delaying the peak pandemic effects and preventing the overflow and subsequent collapse of healthcare systems.^2,3^ Although this pandemic has been widely reported on and discussed for several months, healthcare systems seems to be ill prepared, putting healthcare professionals at the center of complex personal, professional, and societal demands.^1,3-5^ Under extreme pressure, many healthcare professionals may express various psychological problems, such as exhaustion, fatigue, burnout, anxiety, and depression, all of which can undermine their ability to care for their health and safety, as well as that of their colleagues, their close ones, and their patients.^4,5^ Although this problem is especially prevalent among “frontline” healthcare professionals, other professionals may be affected as the pandemic spreads and affects competing interests pertaining to non–pandemic-related health issues.^4,5^ Many proposals have been put forth regarding how to prevent and mitigate these possible adverse effects, such as the introduction of different procedures and guidelines, the availability of instrumental and psychological support, and more appropriate staffing and shift scheduling.^5-8^

As the consequences of advanced pandemics become more evident, even with certain restrictive public health measures in place, the continuity of care for other health issues must be preserved because disrupted availability and access to care in pandemic settings may drive non–pandemic-related mortality.^2,6,9^ Therefore, it is vitally important to control the introduction and spread of SARS-CoV-2 in all healthcare units and institutions that provide care for noninfected patients, so they remain COVID-19 free.^6,8,9^ The emergence of infection in these settings may be particularly difficult to deal with because it may affect vulnerable populations as it rapidly continues to spread.^2,6-9^ Furthermore, this pandemic may further disrupt the availability of care for nonpandemic conditions.^3,6^ This factor is of special relevance in units and institutions dealing with health conditions considered significant risk factors for severe COVID-19 disease.

In these units, among other widely used measures (eg, vigorous screening for possible COVID-19 infection, stringent testing, and triage of suspected cases as well as developing and adopting different care, safety, allocation, and communication strategies), it is of immense importance in controlling possible transmission of infection among and by healthcare professionals. Healthcare professionals may remain unaware of their COVID-19 status due to the mild or even asymptomatic course in healthy individuals; thus, they may unknowingly become supervectors.^6-8,10^ Healthcare settings are usually inadequately prepared for infection prevention and control, especially in the context of limited resources (eg, personal protective equipment), and professionals may become infected through their contact with even seemingly noninfected patients. In addition to previously reported measures, alternative distribution of working schedules may contribute to minimizing the likelihood of virus transmission.

In centers where infected patients are treated, healthcare staff are usually organized in 2-week shifts and then spend the next 2 weeks in self-isolation, preferably being (re)tested before starting a new shift. This approach to staff scheduling seems logical because it follows the COVID-19 incubation period.

Work schedules in “COVID-free” institutions should be organized such that after 1 shift (preferably a 12-hour or, exceptionally, a 24-hour shift), healthcare staff remain in self-isolation for 48 hours. Such a redistribution of shifts makes it possible to resolve the nature of possible infective exposure because the COVID-19 infection window seems to be 48 hours (ie, patients become contagious 48 hours before the onset of symptoms). If a healthcare professional comes into close contact with a patient who later develops symptoms, it is prudent to automatically prolong the self-isolation period until the COVID-19 status of that patient is resolved, allowing enough time to contain possible events of disease transmission. Such a redistribution of working hours, together with rigorous tracking of any relevant close contacts (which could be assisted by novel technologies) limits possible introduction and spread of infection within the institution.

This approach may have special importance in the pandemic timeline. When measures of self-isolation and/or quarantine are in place and local disease transmission has been demonstrated, clinical spread of infection will become the most important. Furthermore, since healthcare professionals among the few persons allowed free movement under “stay at home” orders, this scheduling strategy may have important repercussions for the entire community because it can limit the potential pandemic vector effect, which not yet well understood.^2,6,10^

This scheduling approach may be feasible in institutions with sufficient staff to maintain a work schedule of continuous rotation, which may be difficult, but it may prove useful in the long run. Additionally, through alternative methods of care delivery, like those provided through novel digital technologies, staff that are not physically present may remain fully involved in providing care for those who need it most. Finally, this scheduling approach may preserve the bulk of physically and mentally healthy staff sorely needed to combat later effects of pandemic, which will have dire consequences for healthcare systems that have not made every effort to prevent intrainstitutional transmission.
